# An Unusual Type of Localized Hypertrophic Cardiomyopathy With Wolf Parkinson White Syndrome Presenting With Pulmonary Edema

**DOI:** 10.4021/cr138w

**Published:** 2012-05-20

**Authors:** Mehmet Bulent Vatan, Huseyin Gunduz, Safiye Gurel, Ibrahim Kocayigit, Ahmet Vural, Saadet Demirtas, Mehmet Akif Cakar, Yasemin Gunduz

**Affiliations:** a Sakarya University, Medical faculty, Deparmet of Cardiology, Sakarya, Turkey; b Abant izzet Baysal University Bolu Medical faculty, Deparmet of Radiology, Bolu, Turkey; c Kocaeli University, Medical faculty, Deparmet of Cardiology, Kocaeli, Turkey; d Sakarya University, Medical faculty, Deparmet of Radiology, Sakarya, Turkey

**Keywords:** Hypertrophic cardiomyopathy, Wolf parkinson white syndrome

## Abstract

Hypertrophic cardiomyopathy (HCM) is an autosomal dominant heart disease that is the most common genetic cardiac disorder. The disease is characterized by excessive thickening of the left ventricular myocardium. The anterior portion of the interventricular ventricular septum is often involved. Asymmetric hypertrophy of apical site, left ventricular free wall, and right ventricle are less common in hypertrophic cardiomyopathy that occur in 1% cases. We report a case of a patient with an unusual type of hypertrophic cardiomyopathy and Wolf Parkinson White (WPW) presenting with pulmonary edema.

## Introduction

Hypertrophic cardiomyopathy (HCM) which occurs at a prevalence of one case per 500 population (0.2%), is characterized by an asymetrical wall thickening of the heart that can lead to severe cardiac problems such as progressive heart failure, embolic stroke, and sudden cardiac death [1]. The extent and localization of the wall thickening is highly variable. The interventricular septum is often involved. Asymmetric hypertrophy without involvement of interventricular septum is less common which occurs in 1% cases [2]. We report a case of a patient with an unusual type of hypertropic cardiomyopathy and Wolf Parkinson White (WPW) presenting with pulmonary edema.

## Case Report

A 32-year-old woman was admitted to the emergency room with suddenly occured dispnea and orthopnea. On admission she had a blood pressure of 110/70 mmHg, a regular pulse of 134 beaps per minute, and a breath rate of 34 per minute with 68% of partial oxygen saturation. There was a grade 2 midsystolic murmur in the mesocardiac region and bilateral pulmonary ralles throughout the lung fields.

**Figure 1:**
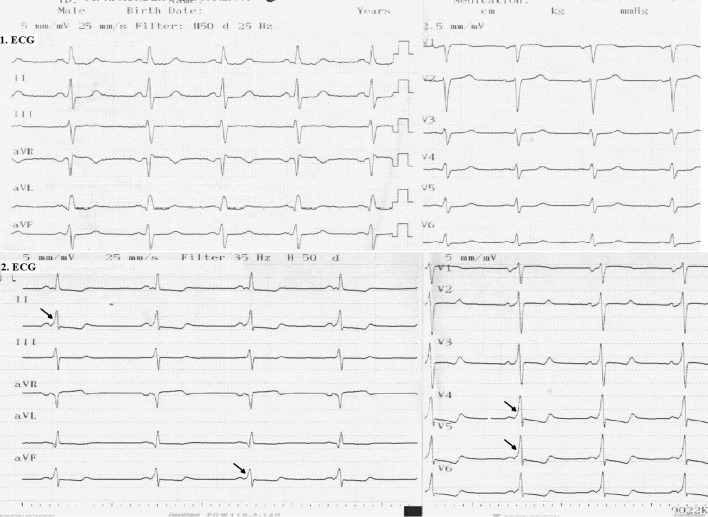
Twelve-lead ECG . The first admission ECG showed sinusal tachycardia. (A). The second admission ECG showed short PR interval and delta wave*(B). ECG: electrocardiogram *Arrows indicates delta waves.

**Figure 2:**
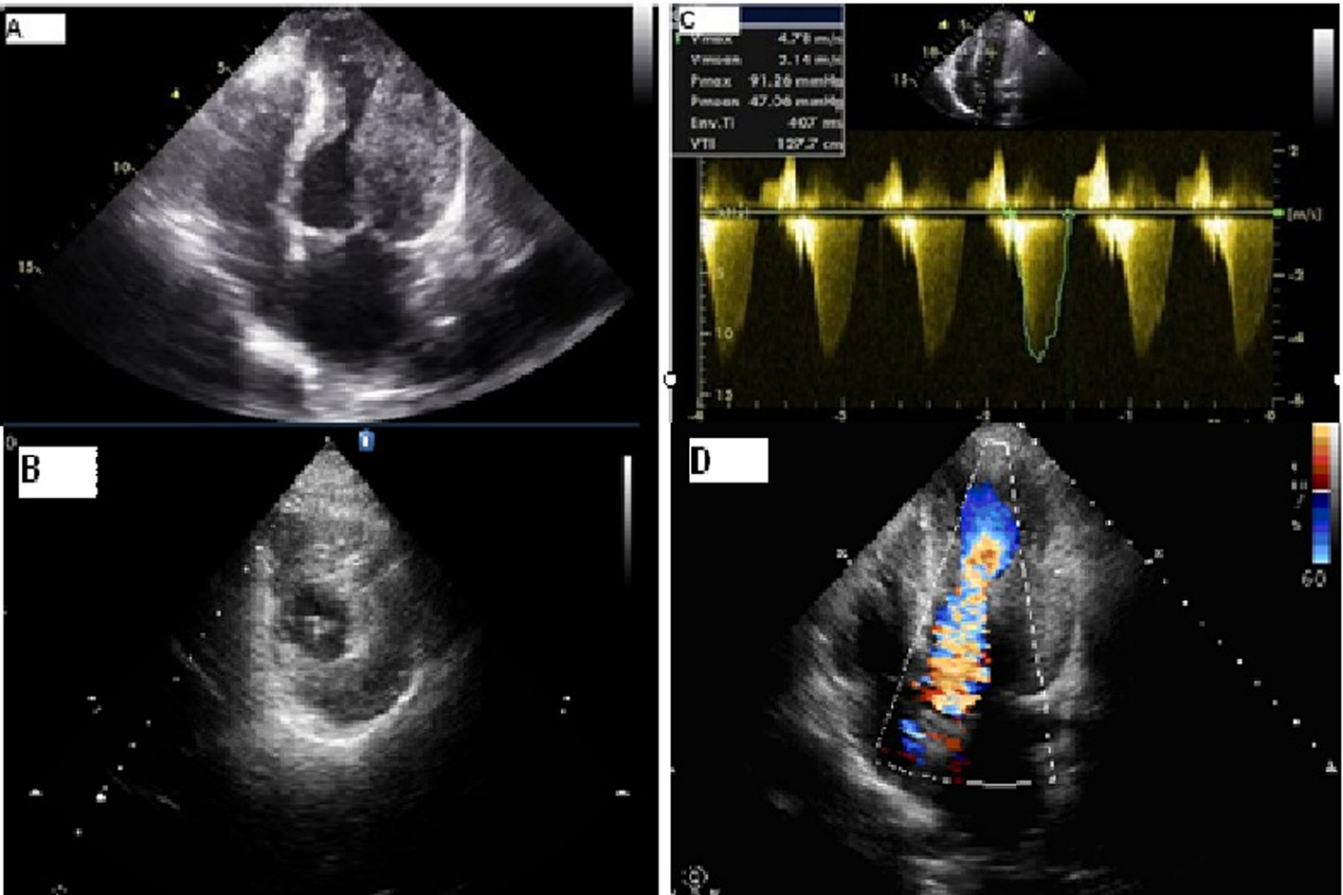
Two-dimensional echocardiogram. Apical four chamber view shows severe wall thickness of the lateral free wall and mid ventricular septum (A). Parasternal short axis view shows severe wall thickness of the posterior and lateral left ventricular free wall, and mid ventricular septum (B). Continuous-wave Doppler study demonstrating increased velocity in the mid ventricular section (C). Color doppler imaging demonstrating flow acceleration due to mid ventricular obstruction (D).

**Figure 3:**
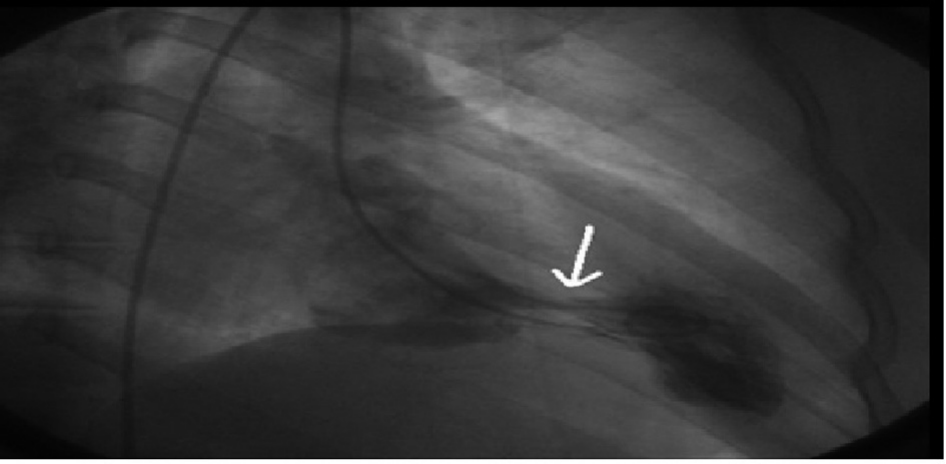
Left ventriculography showing mid ventricular obstruction during systolic phase.

**Figure 4:**
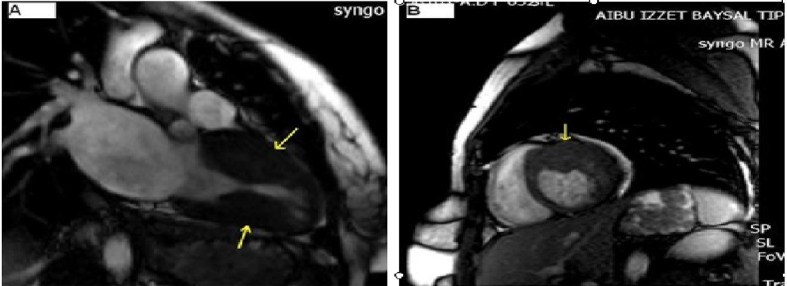
Cardiac MRI imaging. Asymetric hypertrophy of the left ventricle posterior free wall and mid ventricular septum (A) and posterior-lateral free wall (B).

The electrocardiogram (ECG) revealed sinus tachycardia (Fig. 1A). Chest X-ray showed bilateral extended hilar and paranchimal infiltration and lines of Kerley B. The patient was dramatically rapidly improved with diuretic therapy and partial oxygen saturation increased up to 95%. Transthoracic echocardiography (TTE) showed normal left ventricle ejection fraction with severe hypertrophy of left ventricular posterior, lateral free wall, and mid ventricular septum (Fig. 2 A, B). The maximal wall thickness of posterior free wall and mid ventricular septum were 25 mm and 19 mm respectively. There was a significant peak intraventricular gradient of 91 mmHg at rest (Fig. 2C). The color doppler imaging demonstrated flow acceleration due to mid ventricular obstruction (Fig. 2D). The coronary angiography performed because of elevated troponin I levels, that was demostrated normal coronary arteries. Left ventriculography showed totally midventricular obtruction at systolic phase (Fig. 3). The cardiac MRI was also performed to confirm the diagnosis. The cardiac MRI images were clearly showed the asymetric thickening of the left ventricular posterior, lateral free wall, and mid ventricular septum (Fig. 4). The 24 hour Holter ECG monitoring performed and any significant arrhythmias was detected. She was discharged with the prescription of metoprolol and acetyl salisilic acid. After one month of discharge she was admitted to the emergency room with palpitation. The ECG showed atrioventricular nodal reentrant tachycardia. The rhythm was converted to sinus after the administration of metoprolol. The control ECG revealed short PR interval and delta waves as the findings of Wolf Parkinson White syndrome which is different from the ECG on the first admission to the hospital (Fig. 1B). The electrophysiologic study performed and radiofrequency ablation of accessory pathway was applied. Two weeks after hospital discharge, she was admitted to our hospital with ventricular tachycardia which was converted to sinus rhythm after electrical cardioversion. According to the European Society of Cardiology guidelines, ICD implanted because of the risk of sudden death.

## Discussion

Hypertrophic cardiomyopathy (HCM) is one of the most common genetically transmitted cardiovascular disease, and is a leading cause of sudden death amoung adults [1]. The characteristic morphologic feature is asymmetric septal hypertrophy; but asymmetric thickness of apical segments or left ventricular free wall are rare types of HCM [2]. The diversity of the morphologic expression of HCM were due to racial, genetic factors, and differences in phenotypic expression [2]. Andreini et al; described a very atypical form of HCM. In this case the abnormal myocardial thickness was localized to the anterior and lateral free wall of the left ventricle without any involvement of interventricular septum [3]. Duncan et al; described second case of unusual form of HCM with massive midventricular obstruction and an akinetic apical chamber [4]. Lewis et al; described a subgroup of 17 patient with hypertrophic cardiomyopathy, and an unusual and distinctive patern of left ventricular hypertrophy characterized on echocardiography by marked thickening of the posterior free wall and virtually normal or modest increased ventricular septal thickness [5].

Although echocardiography is the tool choice for the diagnosis of HCM, maximal wall thickness and determination of the left ventricular muscle mass can also be determined using cardiac MRI. Myocardial scar tissue can be detected using delayed-enhancement cardiac MRI [6].

Wolf Parkinson White (WPW) syndrome is the most common congenital cardiac abnormality results from an anomalous atrioventricular conduction pathway that can produce ventricular preexcitation and paroxysmal reentrant tachyarrhythmias. The occurence of WPW syndrome is very rare in patients with HCM. It is estimated that 5-10% of patient with HCM are reported to have ventricular preexcitation. The relationship between HCM and WPW syndrome has not clear yet, but there are some clinical evidence to suggest that development of ventricular preexcitation in individuals with HCM may reflect a distinct genetic etiology [7]. A less common type of HCM, known as metabolic HCM that is typically associated with ventricular preexcitation (WPW), is not related to defects in the cardiac sarcomere, but due to glycogen accumulation in cardiac myocytes [8]. There are a few reports about the co-occurrence of HCM and WPW syndrome. Firstly, Shibata et al; described two patients with familial HCM associated with WPW syndrome who showed progression to left ventricle dilatation [9]. Bobkowski at al; reported a case of HCM with asymmetric hypertrophy of ventricular septum and left ventricular posterior wall that was associated with WPW syndrome [10].

To our knowledge this is the first report; the asymetric hypertrophy was limited to the posterior and lateral free wall, and mid ventricular septum in patient with HCM and WPW with acute pulmonary edema. In our case midventricular obstruction and suspected paroxysmal tachyarryhtmia can be responsible from the development of acute pulmonary edema. Since no linkage between the atypical form of HCM with WPW syndrome has been showed in case reports published to date, we cannot conclude whether the prevalance of WPW syndrome is higher in patient with atypical morphologic feature of HCM or an incidental co-occurence of atypically localized HCM and WPW syndrome.
